# Differential effects of clopidogrel and/or aspirin on the healing of tooth extraction wound bone tissue

**DOI:** 10.3389/fphys.2024.1387633

**Published:** 2024-07-17

**Authors:** Jiaping Wang, Juan Lin, Xin Song, Mengting Wang, Yan Chen, Ning Luo, Xin Wu

**Affiliations:** ^1^ Nanjing First Hospital, Nanjing Medical University, Nanjing, China; ^2^ Department of Stomatology, Nanjing First Hospital, Nanjing Medical University, Nanjing, China

**Keywords:** clopidogrel, aspirin, tooth extraction wound, osteogenesis, osteoclast, drug combinations

## Abstract

**Introduction:**

A multitude of variables influence the healing of tooth extraction wounds, and delayed or non-healing extraction wounds might complicate later prosthodontic therapy. In this research, we analyzed the effects of systemic clopidogrel and aspirin alone or in combination on the healing of tooth extraction wounds in mice in order to provide experimental evidence for the healing of extraction wounds in patients who are clinically treated with the two medicines.

**Methods:**

7-week-old ICR mice were randomly divided into four groups: control group (CON), clopidogrel group (CLOP), aspirin group (ASP), and clopidogrel combined with aspirin group (CLOP + ASP); left upper first molar was extracted, after which mice in 1 week of adaptive feeding, CLOP/ASP/CLOP + ASP groups were respectively administered with clopidogrel (10 mg/kg/d), aspirin (15 mg/kg/d), clopidogrel (10 mg/kg/d)+aspirin (15 mg/kg/d), and the control group was given an equal amount of 0.9% saline by gavage. Mice in each group were euthanized at 14 and 28 days postoperatively, and the maxilla was extracted. The tissues in the extraction sockets were examined using MicroCT and sectioned for HE staining, Masson staining, and TRAP staining, and immunohistochemistry staining (for TRAP, RANKL and osteoprotegerin).

**Results:**

MicroCT analysis showed that at day 14, BS/BV was significantly lower in CLOP and CLOP + ASP groups compared to control and ASP groups, while BV/TV, Tb.Th was significantly higher. At day 28, BV/TV was significantly higher in the CLOP + ASP group compared to the CLOP group, with *p* < 0.05 for all results. HE staining and Masson trichrome staining findings revealed that at day 28, the mesenchyme in the bone was further decreased compared to that at day 14, accompanied with tightly arranged and interconnected bone trabeculae. In the quantitative analysis of Masson, the fraction of newly formed collagen was significantly higher in the CLOP group in comparison with that in the CON group (*p* < 0.05). At day 14, the ASP group had substantially more TRAP-positive cells than the CLOP and CLOP + ASP groups (*p* < 0.05). In immunohistochemical staining, RANKL expression was found to be significantly higher in the ASP group than those in the other three groups at day 28 (*p* < 0.05); OPG expression was significantly higher in the CLOP group and the CLOP + ASP group compared with that at day 14, and was higher than that in the ASP group at day 14 and day 28. OPG/RANKL was significantly higher in the CLOP and the CLOP + ASP groups than in the ASP group (*p* < 0.05).

**Conclusion:**

Clopidogrel alone promotes osteogenesis in the extraction wound, whereas aspirin alone inhibits alveolar bone healing. When the two drugs were combined, the healing effect of the extraction wound was more similar to that of the clopidogrel alone group. These results indicated that clopidogrel could promote the healing of the tooth extraction wound, and neutralize the adverse effect of ASP on osteogenesis when the two drugs were used in combination.

## 1 Introduction

Tooth extraction causes alveolar bone resorption, and clinical data showed that the most significant changes in the alveolar ridge have been seen in the immediate post-extraction period (Arioka et al., 2022). Many factors affect the healing and remodeling of the alveolar bone, one of which cannot be ignored is the effect of long-term systemic medication on the healing of the bone tissue.

The prevalence of cardiovascular disease has been rising annually in recent years (Yang et al., 2016). The common antiplatelet medications aspirin (also known as acetylsalicylic acid) and clopidogrel (also known as Plavix) are used extensively for the treatment and prevention of cardiovascular illnesses (Dehmer et al., 2016; Hwang et al., 2023). Aspirin is an inhibitor of prostaglandin synthesis (Franceschi and Campisi, 2014). Clopidogrel is a thienopyridine analogue that inhibits platelet function by irreversibly inhibiting P2Y12 adenosine diphosphate (ADP) receptors (Jørgensen et al., 2012), both of which have the potential to affect bone metabolism (Mao et al., 2022). The amount and density of new bone in the alveolar socket can affect the success of subsequent treatment of prosthodontics (Hämmerle et al., 2012; Rezende et al., 2017). For example, the success rate of oral implant surgery and the long-term stability of implants are closely related to the amount and density of bone at the implant site (Ersanli et al., 2004).

Generally, aspirin and clopidogrel, either alone or in combination, need to be taken for an extended period, and patients who require dental extractions need to continue to take the antiplatelet drugs for systemic disease during the healing process of the tooth extraction trauma, regardless of whether the medication is discontinued before or after the extraction (Sadeghi-Ghahrody et al., 2016). Interestingly, the effect of aspirin and clopidogrel, alone or in combination, on the healing of extraction socket bone tissue remains unknown. The healing of extraction sockets has a unique environment (Ito et al., 2022). Therefore, in this study, the mouse models of tooth extraction was constructed (Vieira et al., 2015). The effects of aspirin and clopidogrel, either alone or in combination, on the healing of extraction sockets in mice were investigated using imaging, histology, and immunohistochemistry. This study aims to determine how aspirin, clopidogrel, and their combination affect the healing of bone tissue in tooth extraction wounds, so as to provide a reference for the prognosis of tooth extraction and the following prosthodontics treatment for patients taking these two medications in clinical practice.

## 2 Materials and methods

### 2.1 Establishment of animal models

All experimental animals were purchased from Zhejiang Vital River Laboratory Animal Technology Co. Ltd. and male 7-week-old healthy ICR mice (body weight 20 g±3 g) were selected for the study after 1 week of adaptive feeding. The Ethics Committee of Nanjing Medical University (Nanjing, China) approved the study’s ethical components (permission number: DWSY-23013317) and were housed in the Animal Experiment Centre of Nanjing Hospital affiliated to Nanjing Medical University, in which the animal rooms were air-conditioned and maintained at 22°C–24°C, with alternating light and dark cycles every 12 h (light cycle from 7:00 a.m. to 7:00 p.m.). Mice had free access to tap water at all times.

After a week of acclimation and feeding, the experimental mice received an intraperitoneal injection of 1% sodium pentobarbital for general anesthesia. Following anesthesia, the first molar of the left maxillary was extracted. Four groups of sixteen mice each were randomly assigned: the control group (CON), the clopidogrel group (CLOP), the aspirin group (ASP), and the clopidogrel plus aspirin group (CLOP + ASP). Drugs and dosages: 0.9% saline solution as solvent control, 10 mg/kg clopidogrel (MedChemExpress HY17459 United States), 15 mg/kg aspirin (MedChemExpress HY14654 United States), 10 mg/kg clopidogrel +15 mg/kg aspirin, administered by gavage once daily. The dosing cycles were 14 days and 28 days. During the administration period, the solid feed was soaked and softened for mice. After the 14th and 28th days of the administration, eight mice in each group were euthanized, and their heads were fixed in 4% paraformaldehyde solution (for 24 h) for further analysis.

### 2.2 Micro-CT analysis

After fixation of the mouse head in 4% paraformaldehyde solution for 24 h, high-resolution X-ray 3D imaging of the tooth extraction wound area of the left alveolar bone of the mouse was performed without destroying the samples using a high-resolution Micro-CT system (SkyScan 1176 Bruker, Germany), scanning of the samples was performed at 50 kV and 455 μA with Exposure (ms) = 265 and voxel resolution of 18 µm. The raw data were obtained layer by layer, and 3D reconstruction was performed to analyze the extraction sockets analysis of the Bone surface/volume ratio (BS/BV), Percent bone volume (BV/TV), Trabecular thickness (Tb.Th), and the Trabecular number (Tb.N), amongst other parameters were analyzed.

### 2.3 HE staining

Histological staining After Micro-CT scanning, the left half of the maxilla of each animal was slowly decalcified in 10% ethylenediaminetetraacetic acid (EDTA) (pH 7.4) for 2 months. All specimens were then dehydrated in a series of alcohol baths and embedded in paraffin. Maxillary alveolar fossa samples were cut into 5 μm thick sections along the sagittal plane direction, stained with hematoxylin (Freethinking FS141500), washed in tap water, alcohol fractionated in 1% hydrochloric acid, rinsed in tap water, stained with eosin stain (FS142500), rinsed in tap water, and dehydrated and sealed.

### 2.4 Masson staining

The samples were decalcified and sectioned, hematoxylin stained, washed in tap water, differentiated in 1% acid alcohol, rinsed in tap water, rinsed in running water to return the blue color, stained in Lichtenstein’s red acidic magenta solution, slightly washed in distilled water, treated with aqueous phosphomolybdic acid, restained in aniline blue solution, treated with 0.2% glacial acetic acid and dehydrated to seal the slices. HE staining and Masson trichrome staining (Freethinking FH115100) were performed for histological analysis, which was used to observe the tissue changes during the healing process of extraction wounds.

### 2.5 TRAP staining

TRAP staining was performed with an anti-tartrate acid phosphatase staining kit (TRAP kit) (Servicebio, Wuhan, GR2107063) to observe the osteoclast changes. Paraffin sections were deparaffinized into distilled water, incubated at 37°C in distilled water, then incubated by TRAP staining solution at 37°C for 1 h, rinsed in distilled water, counterstained with hematoxylin on slides, rinsed in distilled water, differentiated by 1% hydrochloric acid in alcohol, counterstained in counter blue solution, washed in water, and then dehydrated and sealed. Multinucleated TRAP-positive cells located within the extraction wound were recorded as osteoclasts, and the number of osteoclasts was calculated using TRAP staining in this way.

### 2.6 Immunohistochemistry staining for RANKL and OPG

Sections were dewaxed, hydrated, antigenically repaired, cooled to room temperature, and rinsed three times with TPBS; incubated in 3% hydrogen peroxide solution at room temperature, protected from light, and washed with shaking on a decolorizing shaker; serum blocking solution was added dropwise and incubated at room temperature; primary anti-OPG (Affinit dilution of 1:150; DF6824) and RANKL (Affinit dilution of 1:150, AF0313) were added, and the slices were flatly placed in a wet box and incubated overnight at 4°C; horseradish peroxidase-labeled antibody II was added; sections were slightly shaken dry and freshly prepared DAB development solution (Freethinking) was added dropwise in a circle. Control the time of chromogenic development under a microscope; rinse the slices with tap water to terminate the chromogenic development. The sections were washed with tap water to stop the color development.

All above-stained sections were observed under the 200 field of view of an Olympus BX51 orthostatic microscope.

### 2.7 Statistical analysis

Using CTAN software (version 1.18.8.0, Bruker, Kontich, Belgium), the samples were reconstructed in three dimensions. The volume of interest (VOI) was chosen as the extraction wound area, and measurements of BS/TV, BV/TV, Tb.Th, and Tb.N were obtained.

All graphs were carefully generated and analyzed using GraphPad Prism 9 (version 9.5.1 San Diego, CA, United States), and values are expressed as mean ± standard. When faced with homogeneity of variance, comparisons between multiple groups were made using one-way ANOVA (followed by Tukey’s *post hoc* test). Welch’s ANOVA was introduced when the variance was heterogeneous, followed by the Games Howell test. A value of *p* < 0.05 was considered a statistically significant difference.

## 3 Results

### 3.1 CLOP and CLOP + ASP promoted alveolar bone healing according to radiographic findings

**FIGURE 1:**
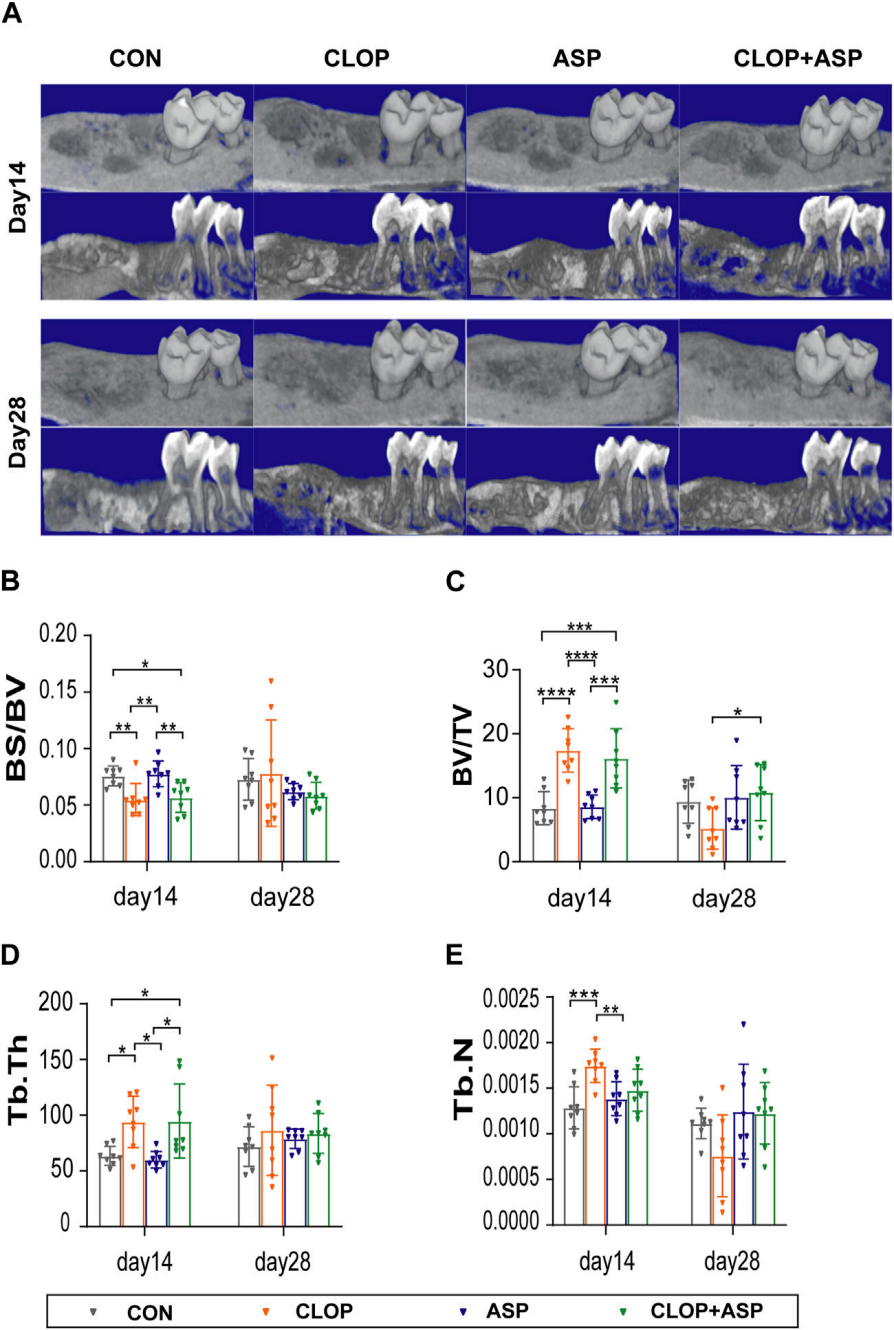
Radiographic analysis of tooth extraction wound. **(A)** Micro-CT and three-dimensional (3D) reconstruction images showed the new bone formamation in tooth extration. **(B–E)** Parameter analysis of tooth extration. Parameters including bone surface/volume ratio (BS/BV), percent bone volume (bone volume/tissue volume BV/TV), trabecular thickness (Tb.Th)and, trabecular number (Tb.N) were examined on day 14 and day 28. Data are shown as mean ± SD, n = 8/group. *p < 0.05, **p < 0.01 (one-way ANOVA followed by Tukey’s multiple comparisons).

The 3D reconstruction of the extraction wound’s bone tissue using MicroCT and analysis to find each parameter (Figures 1A–E) revealed that, on day 14, the CLOP and CLOP + ASP groups exhibited a significantly lower BS/BV ratio than the CON and ASP groups (Figure 1B. *p* < 0.05); the CLOP and CLOP + ASP groups had a significantly higher BV/TV, Tb.Th ratio than the CON and ASP groups (Figures 1C,D. *p* < 0.05); the CLOP group had a higher Tb.N ratio than the CON and ASP groups (Figure 1E. *p* < 0.05). After a 28-day administration, all the parameters in the control group showed no statistically significant variations, with the exception of BV/TV, which was significantly greater in the CLOP + ASP group than in the CLOP group (Figure 1B. *p* < 0.05). The findings mentioned above showed that aspirin prevented alveolar bone healing at the corresponding supplied levels during the healing process of an extraction wound, but clopidogrel enhanced bone tissue healing. When clopidogrel and aspirin were combined, the results were more favorable to the facilitator effect of clopidogrel alone.

### 3.2 Clopidogrel promoted the formation of bone collagen in alveolar socket

**FIGURE 2:**
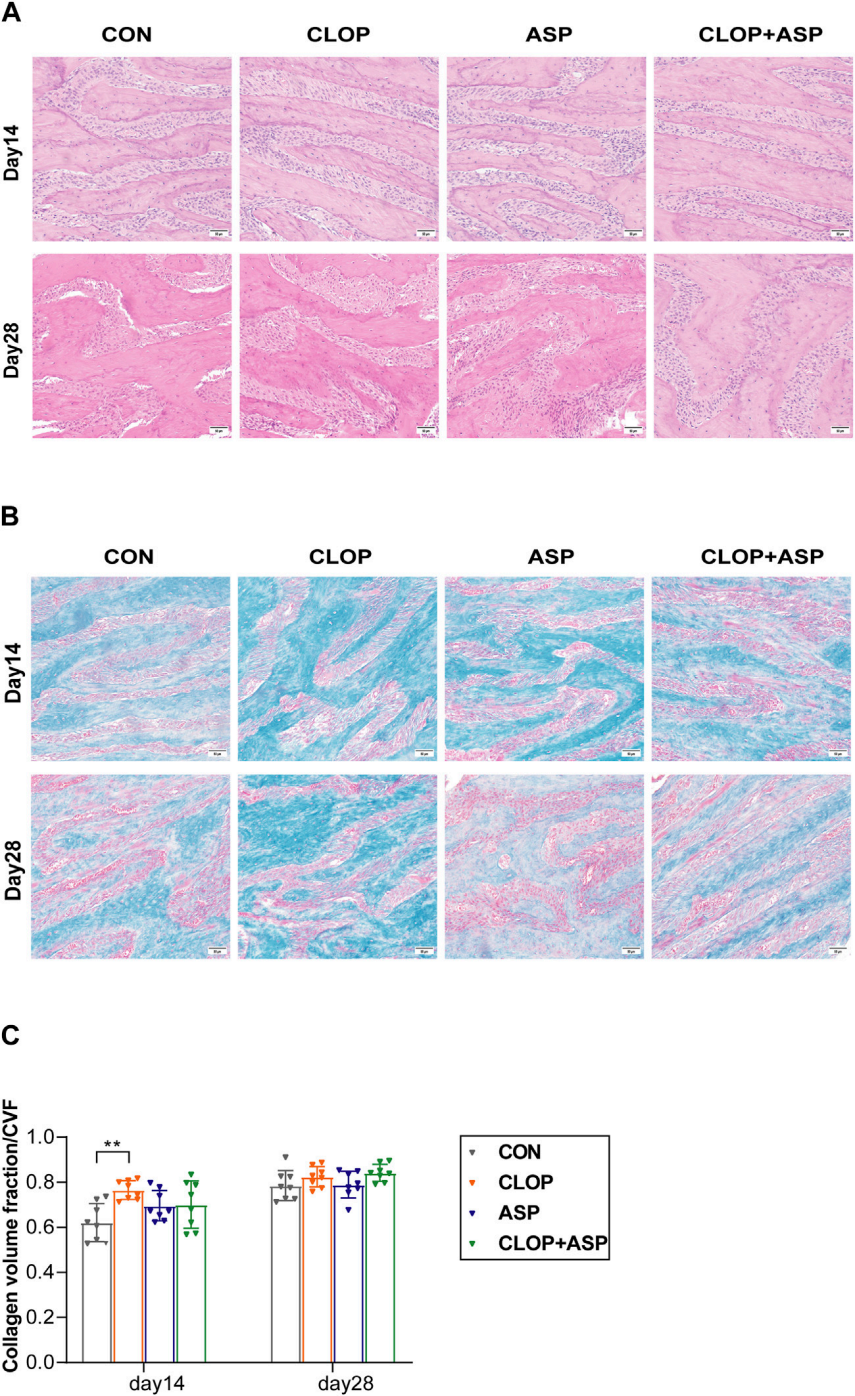
Histological changes of tooth extraction. **(A)** HE staining of new bone. Photomicrographs illustrating the histological findings in the control group (CON), clopidogrel group (CLOP), aspirin group (ASP) and clopidogrel and aspirin group (CLOP + ASP) at day 14 and day 28. Scale bar = 50 μm. **(B)** Masson’s trichrome staining. Scale bar = 50 μm. **(C)** Quantitative analysis of newly formed collagen in the extraction sockets. Data are shown as mean ± SD, n = 8/group. *p < 0.05, **p < 0.01 (one-way ANOVA followed by Tukey’s multiple comparisons).

Tissue healing in the extraction wound was examined using HE staining and Masson’s trichrome staining. On day 14, the alveolar socket was filled with new bone, but the trabecular arrangement was more disorganized than it was on day 28. On day 28, the mesenchyme in the bone was further reduced, and the trabeculae were tightly connected (Figure 2A). Masson staining showed that more new bone was arranged disorderly and stained blue into cords on day 14. It was statistically significant that the CLOP group produced considerably more collagen than the CON group did. Blue-stained new bone was organized into a network on day 28, and there was a noticeable rise in the quantity of new bone (Figure 2B). The CVFs of the CLOP and CLOP + ASP groups were more significant than the CON group’s (Figure 2C), although there was no statistically significant difference.

### 3.3 ASP stimulates osteoclast activity while CLOP and CLOP + ASP groups suppress osteoclast activity in the healing phase of extraction wounds

**FIGURE 3:**
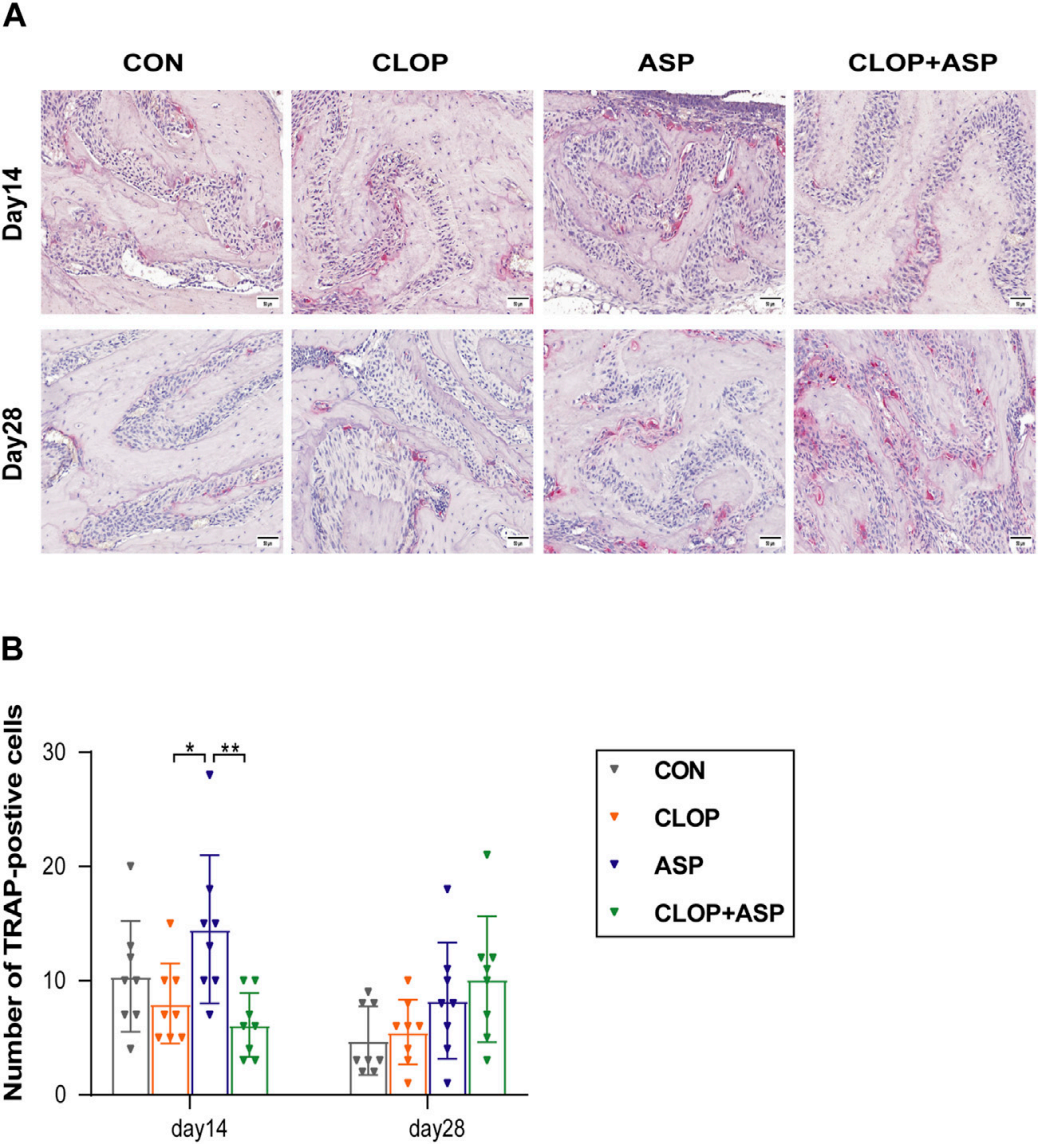
TRAP staining. **(A)** TRAP-positive osteoclasts were detected in each group of tooth extraction. Scale bar = 50 μm. **(B)** Quantification of TRAP-positive cells. Data are shown as mean ± SD, n = 8/group. *p < 0.05, **p < 0.01 (one-way ANOVA followed by Tukey’s multiple comparisons).

**FIGURE 4:**
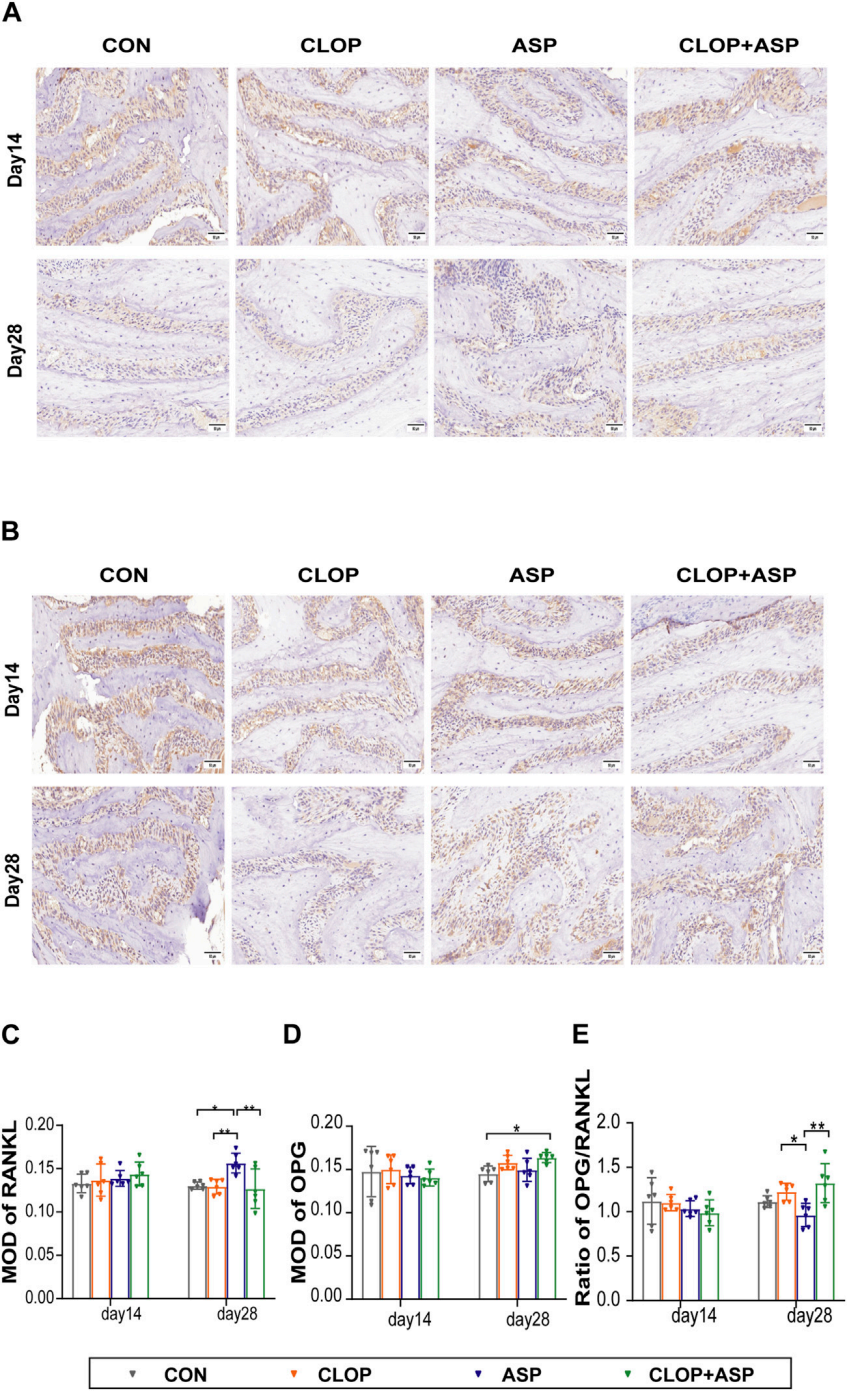
Immunohistochemical (IHC) staining of RANKL and OPG of tooth extration. **(A)** IHC staining of RANKL of tooth extration. Scale bar = 50 μm. **(B)** IHC staining of OPG of tooth extration. Scale bar = 50 μm. **(C–E)** The means of the integrated optical density (MOD) of RANKL, OPG, and OPG/RANKL ratio were shown. Data are shown as mean ± SD, *n* = 6/group. *p < 0.05, **p < 0.01 (one-way ANOVA followed by Tukey’s multiple comparisons).

The analysis focused on the activity of osteoclasts in the alveolar fossa as the tissue from the extraction site healed. To find the percentage of TRAP-positive cells in the CON, CLOP, ASP, and CLOP + ASP groups, TRAP staining was performed (Figure 3A). On day 14, the ASP group had a significantly more significant number of TRAP-positive cells (*p* < 0.05) than the CLOP and CLOP + ASP groups. On day 14 and 28, however, there was no statistically significant difference between the drug and control groups (Figure 3B). We measured the expression levels of OPG and RANKL. The primary differentiation of RANKL occurs from osteoblasts and their immature precursor cells. Positive cells had blue nuclei and a yellowish-brown extracellular matrix staining due to cytoplasmic RANKL staining (Figure 4A). On day 28, the ASP group’s expression was considerably greater than the expressions of the other three groups (Figure 4C. *p* < 0.05). OPG is primarily a significant glycoprotein that is released and made by osteoblasts. OPG can bind to RANKL, inhibiting osteoclast maturation and differentiation and stopping excessive bone resorption. OPG staining is cytoplasmic. The nucleus of positive cells is light blue, and the extracellular matrix has a yellow-brown staining (Figure 4B).In this study, OPG expression was significantly higher in the CLOP and CLOP + ASP groups on day 28 compared to day 14 and higher than in the ASP group; on day 28, the CLOP + ASP group’s expression was significantly higher than that of the CON group (Figure 4D. *p* < 0.05). On day 28, the CLOP and CLOP + ASP groups had significantly greater OPG/RANKL than the ASP group (Figure 4E. *p* < 0.05). Because OPG inhibits bone resorption, higher OPG/RANKL ratios indicate reduced bone resorption and are often associated with osteogenesis.

## 4 Discussion

There were four groups in this study: CON, CLOP, ASP, and CLOP + ASP, which was a two-drug combination group. Results indicated that, compared to the CON group, the CLOP group facilitated the healing of extraction wounds. When comparing the ASP group to the CON group, the ASP group prevented extraction wound healing. The CLOP + ASP group was also set up in this study, and the results showed that the combination group was close to the CLOP group alone, which promoted the healing of extraction wounds. When aspirin and clopidogrel are combined at this dosage, the effect of clopidogrel on bone metabolism is greater than that of aspirin, which corrects the negative effect of aspirin on bone metabolism.

Aspirin is a non-steroidal anti-inflammatory drug (NSAID) with several drug mechanisms, including i) it is a non-selective cyclooxygenase (COX) inhibitor, which inactivates COX acetylation through irreversible inhibition of cyclooxygenase (COX), and ii) reduction of prostaglandin (prostaglandin, PG) synthesis, PGE2 is involved in signal transduction between osteoblasts and is essential for differentiation and expression of osteoblasts and osteoclasts (Blackwell et al., 2010) and iii) can affect the production of nitric oxide (NO) and NF-κB, and all of the above mechanisms of action can affect bone metabolism (Carbone et al., 2003). Vestergaard et al. (2012) conducted a population-based epidemiological study in a Danish population to assess the relationship between platelet inhibitor use and fracture incidence. The researchers discovered that clopidogrel did not raise the risk of fracture and that low-dose aspirin did. The authors came to the conclusion that more study is required to ascertain if platelet inhibitors may have an impact on bone metabolism *in vivo*. Mao and colleagues (Mao et al., 2022) examined how the duration and dosage of clopidogrel and aspirin, either separately or together, affected the risk of hip fractures in individuals with type 2 diabetes mellitus, selecting patients with newly diagnosed T2DM and dividing them into an aspirin monotherapy group (78,522 patients), a clopidogrel monotherapy group (12,752 patients), a doublet therapy group (7,209 patients), and a group that did not take antiplatelet drug group (401,686 cases). The authors found that only higher doses and longer durations of antiplatelet medication (≥3 years) reduced fracture incidence and that lower doses and shorter durations (1–2 years) were even associated with a higher risk of fracture; this clinical study specifically included a combination group, and the results suggest that dual antiplatelet medication does affect bone health. Chen et al. (2011) gave 8.93, 26.79, and 80.36 mg/kg of aspirin daily to 3-month-old ovariectomized rats in order to investigate the therapeutic benefits of aspirin on postmenopausal osteoporosis. They demonstrated that aspirin’s effects on bone were dose-dependent. At day 14, the CLOP group in the current study was considerably higher than the ASP group in BV/TV, Tb.Th, and Tb.N, and significantly lower than the ASP group in BS/BV, according to the data displayed by MicroCT. The ASP group inhibited the healing of extraction wounds compared to the CON and CLOP groups. Depending on the epidemiological studies, different animal models, different dosages administered and maintenance time, the effect of aspirin on human bone health, and more specifically, the effect of aspirin on alveolar bone healing, needs to be supported by more research data.

Clopidogrel is a thienopyridine analogue that irreversibly inhibits the P2Y12 ADP receptor (Jørgensen et al., 2012). Both osteoblasts and osteoclasts have P2Y12 ADP receptors. It was found that clopidogrel reduced the release of alpha and dense granules from platelets by irreversibly binding to the P2Y12 receptor (Garcia et al., 2011) and inhibited the binding of β-3 integrins (Lindemann et al., 2001). The β-3 integrin plays a crucial role in osteoclast formation, adhesion, and bone resorption (Feng et al., 2001). Clopidogrel can act directly on osteoclasts, interfering with osteoclast differentiation and activity, and its effects on bone metabolism depend on different degrees of inhibition (Jørgensen et al., 2012; Mao et al., 2022; Coimbra et al., 2014 constructing an experimental periodontitis animal model, investigated the effects of aspirin and clopidogrel on mesenchymal stem cells in rats with periodontitis. The administration group was given aspirin or clopidogrel daily by gavage, and the animals were executed for three consecutive days to observe the inflammatory process of periodontal disease. It was detected that clopidogrel, but not aspirin, had a preventive effect against bone loss when aspirin and clopidogrel were administered systemically. It was thought that clopidogrel could directly interfere with osteoclast differentiation and activity. Clop’s bone protection in periodontal disease models requires further mechanistic exploration, which may provide insights into new clinical treatment strategies. Coimbra et al., 2015, in studying the effects of aspirin and clopidogrel on mesenchymal stem cells in periodontitis in rats, found that clopidogrel was effective in reducing inflammatory infiltration and increasing the number of osteoblasts and mesenchymal stem cells and had a positive effect on mesenchymal stem cells when repair had already been initiated, whereas aspirin had no significant effect.

Clopidogrel may play a positive role in the tissue repair process. Lillis et al. investigated whether continuous perioperative treatment with clopidogrel had a negative effect on bone healing in a rabbit model of cranial defects. Two circular defects were created in the skulls of 16 male New Zealand White rabbits, which were randomly divided into two groups: one group was given clopidogrel 3 mg/kg per day for 1 week preoperatively, and the other was given only the lysogenic, treatment was continued for 6 weeks postoperatively, and it was found that clopidogrel promotes bone healing during the perioperative period without negative effects (Lillis et al., 2019). In the present study, according to the results shown by MicroCT, the CLOP group could also promote the healing of the extraction wound bone tissue at 14 days. The effect of the dual-antibody combination group was similar to that of the CLOP group. It also promoted the healing of the extraction wound, which further supports the positive role of clopidogrel in bone tissue repair. Clopidogrel is likely to be therapeutic. This study further supports the positive role of clopidogrel in bone tissue repair and the potential therapeutic value of clopidogrel in the healing of extraction wounds.

The RANKL (RANK ligand)/RANK (NF-κB receptor activator)/OPG (osteoprotegerin) system is essential for bone resorption (Kong et al., 1999). RANK is expressed on osteoclasts and their immature precursor cells. Osteoblasts and their immature precursor cells mainly express RANKL. RANKL binds to RANK, promotes osteoclast differentiation and maturation, and inhibits apoptosis (Ono et al., 2020). OPG is an important antagonistic receptor produced by osteoblasts, which can bind RANKL competitively with RANK, and the competitive binding ability of OPG to RANKL is better than that of RANK, so OPG can delay osteoclast differentiation and maturation and inhibit excessive bone resorption by blocking RANK and RANKL binding (Azizieh et al., 2019). Changes in the OPG/RANKL ratio regulate the differentiation and maturation of osteoclasts, and the RANK/RANKL/OPG system may influence bone resorption by regulating the OPG/RANKL ratio (de Molon et al., 2018). A higher ratio of OPG/RANKL aids in producing new bone, whereas a lower value encourages the resorption of existing bone.

In order to quantify osteoclast activity in the context of clopidogrel and aspirin-assisted extraction wound healing, this study calculated the number of TRAP-positive cells and the OPG/RANKL ratio. TRAP-positive cells were significantly more prevalent in the ASP group on day 14 compared to the other groups. On day 28, it was found that the ASP group had lower levels of OPG/RANKL than the other groups. RANKL and OPG expression levels were assessed by IHC staining. The results suggest that aspirin plays a vital role in osteoclastogenesis at this dose, which contradicts previous experiments (Wei et al., 2015; Xin et al., 2023). On the one hand, this could be connected to aspirin’s dose-dependent effect (Kazui et al., 2010). In this study, The dosages of the mice were adjusted to correspond with the regularly prescribed doses of aspirin 100 mg/d and clopidogrel 75 mg/d for adults in the prevention and treatment of cardiovascular and cerebrovascular disorders in the clinic, and the doses of the mice were changed to match the mouse doses. On the other hand, bone regeneration can be divided into intramembranous ossification and endochondral ossification. The extraction socket is repaired by intramembranous ossification, which lacks a cartilaginous appearance, and the healing process of extraction wounds is differentiated from the healing and repair of long-bone fractures so that future studies will need to determine the mechanisms that regulate these two different modes of ossification (Chen et al., 2011).

In this study, we established an animal model of a healthy extraction socket. We investigated how the combined or individual use of aspirin and clopidogrel affected the repair of bone tissue in the extraction socket. Given that clopidogrel has a positive effect on wound healing, future studies will look at the possible effects of topical therapies, including clopidogrel-loaded biocomposites, on the healing of extraction wounds.

There are some limitations to our study; clopidogrel, as a precursor drug, needs to be metabolized in the liver by a variety of drug-metabolizing enzymes to the sulfhydryl-active metabolite, H4, for its antiplatelet effect to be effective (Kazui et al., 2010; Xie et al., 2011). *In vitro* cellular experiments with clopidogrel active substance lack *in vivo* metabolic processes in the liver, so we constructed animal models in which clopidogrel is metabolized by the animal to act, facilitating the elaboration of the final phenomenon under study. In dental research, mice and other rodent models are commonly employed and are essential for studying the wound healing process following tooth extractions, as well as the impact of medications on this process. This is an emerging animal model (Bodner et al., 1993; Chew et al., 2022). However, considering factors such as bone structure, size, and growth rate, it has been found that extrapolation from mice to humans still needs to consider the differences between the two (Wancket, 2015). Aspirin and clopidogrel are often used in clinical settings to treat many chronic illnesses, provided that confounding variables are appropriately adjusted for. Future clinical research is necessary to explore the impact of dual antagonists on the metabolism of bone tissue.

## 5 Conclusion

In our study, clopidogrel alone was found to promote bone healing in the extraction wound at the set dose of the drug, whereas aspirin alone inhibited alveolar bone healing accordingly. In the clopidogrel combined with the aspirin group, it was found that the healing of the extraction wound tended to be more similar to the clopidogrel alone group in the presence of the combination, i.e., it contributed to the healing of the extraction wound.

## Data Availability

The original contributions presented in the study are included in the article/Supplementary Material, further inquiries can be directed to the corresponding author.

## References

[B1] Arioka M. Dawid I. M. Cuevas P. L. Coyac B. R. Leahy B. Wang L. (2022). Accelerating socket repair via WNT3A curtails alveolar ridge resorption. J. Dent. Res. 101, 102–110. 10.1177/00220345211019922 34157887

[B2] Azizieh F. Y. Shehab D. Jarallah K. A. Gupta R. Raghupathy R. (2019). Circulatory levels of RANKL, OPG, and oxidative stress markers in postmenopausal women with normal or low bone mineral density. Biomark. Insights 14, 1177271919843825. 10.1177/1177271919843825 31452599 PMC6700864

[B3] Blackwell K. A. Raisz L. G. Pilbeam C. C. (2010). Prostaglandins in bone: bad cop, good cop? Trends Endocrinol. Metab. 21, 294–301. 10.1016/j.tem.2009.12.004 20079660 PMC2862787

[B4] Bodner L. Kaffe I. Littner M. M. Cohen J. (1993). Extraction site healing in rats. A radiologic densitometric study. Oral Surg. Oral Med. Oral Pathol. 75, 367–372. 10.1016/0030-4220(93)90153-u 8469551

[B5] Carbone L. D. Tylavsky F. A. Cauley J. A. Harris T. B. Lang T. F. Bauer D. C. (2003). Association between bone mineral density and the use of nonsteroidal anti-inflammatory drugs and aspirin: impact of cyclooxygenase selectivity. J. Bone Min. Res. 18, 1795–1802. 10.1359/jbmr.2003.18.10.1795 14584890

[B6] Chen Z. Wu Z. Sang H. Qin G. Wang L. Feng J. (2011). Effect of aspirin administration for the treatment of osteoporosis in ovariectomized rat model. Zhonghua Yi Xue Za Zhi 91, 925–929. 21600123

[B7] Chew R. J. J. Lu J. X. Sim Y. F. Yeo A. B. K. (2022). Rodent peri-implantitis models: a systematic review and meta-analysis of morphological changes. J. Periodontal Implant Sci. 52, 479–495. 10.5051/jpis.2200900045 36468467 PMC9807853

[B8] Coimbra L. S. Steffens J. P. Alsadun S. Albiero M. L. Rossa C. Pignolo R. J. (2015). Clopidogrel enhances mesenchymal stem cell proliferation following periodontitis. J. Dent. Res. 94, 1691–1697. 10.1177/0022034515598273 26220958 PMC4681474

[B9] Coimbra L. S. Steffens J. P. Muscará M. N. Rossa C. Spolidorio L. C. (2014). Antiplatelet drugs reduce the immunoinflammatory response in a rat model of periodontal disease. J Periodontal Res. 49, 729–735. 10.1111/jre.12155 24372313

[B10] DehmerS. P.MaciosekM. V.FlottemeschT. J.LaFranceA. B.WhitlockE. P. (2016). Aspirin for the primary prevention of cardiovascular disease and colorectal cancer: a decision analysis for the U.S. Preventive services task force. Ann. Intern Med. 164, 777–786. 10.7326/M15-2129 27064573

[B11] de Molon R. S. Park C. H. Jin Q. Sugai J. Cirelli J. A. (2018). Characterization of ligature-induced experimental periodontitis. Microsc. Res. Tech. 81, 1412–1421. 10.1002/jemt.23101 30351474

[B12] Ersanli S. Olgac V. Leblebicioglu B. (2004). Histologic analysis of alveolar bone following guided bone regeneration. J. Periodontol. 75, 750–756. 10.1902/jop.2004.75.5.750 15212358

[B13] Feng X. Novack D. V. Faccio R. Ory D. S. Aya K. Boyer M. I. (2001). A Glanzmann’s mutation in beta 3 integrin specifically impairs osteoclast function. J. Clin. Invest. 107, 1137–1144. 10.1172/JCI12040 11342577 PMC209281

[B14] Franceschi C. Campisi J. (2014). Chronic inflammation (inflammaging) and its potential contribution to age-associated diseases. J. Gerontol. A Biol. Sci. Med. Sci. 69 (Suppl. 1), S4–S9. 10.1093/gerona/glu057 24833586

[B15] Garcia A. E. Mada S. R. Rico M. C. Dela Cadena R. A. Kunapuli S. P. (2011). Clopidogrel, a P2Y12 receptor antagonist, potentiates the inflammatory response in a rat model of peptidoglycan polysaccharide-induced arthritis. PLoS One 6, e26035. 10.1371/journal.pone.0026035 22028806 PMC3196585

[B16] Hämmerle C. H. F. Araújo M. G. Simion M. Osteology Consensus Group 2011 (2012). Evidence-based knowledge on the biology and treatment of extraction sockets. Clin. Oral Implants Res. 23 (Suppl. 5), 80–82. 10.1111/j.1600-0501.2011.02370.x 22211307

[B17] Hwang Y. J. Chang H.-Y. Metkus T. Andersen K. M. Singh S. Alexander G. C. (2023). Risk of major bleeding associated with concomitant direct-acting oral anticoagulant and clopidogrel use: a retrospective cohort study. Drug Saf. 47, 251–260. 10.1007/s40264-023-01388-z 38141156 PMC10942724

[B18] Ito S. Kasahara N. Kitamura K. Matsunaga S. Mizoguchi T. Htun M. W. (2022). Pathological differences in the bone healing processes between tooth extraction socket and femoral fracture. Bone Rep. 16, 101522. 10.1016/j.bonr.2022.101522 35372643 PMC8965168

[B19] Jørgensen N. R. Grove E. L. Schwarz P. Vestergaard P. (2012). Clopidogrel and the risk of osteoporotic fractures: a nationwide cohort study. J. Intern Med. 272, 385–393. 10.1111/j.1365-2796.2012.02535.x 22372976

[B20] Kazui M. Nishiya Y. Ishizuka T. Hagihara K. Farid N. A. Okazaki O. (2010). Identification of the human cytochrome P450 enzymes involved in the two oxidative steps in the bioactivation of clopidogrel to its pharmacologically active metabolite. Drug Metab. Dispos. 38, 92–99. 10.1124/dmd.109.029132 19812348

[B21] Kong Y. Y. Yoshida H. Sarosi I. Tan H. L. Timms E. Capparelli C. (1999). OPGL is a key regulator of osteoclastogenesis, lymphocyte development and lymph-node organogenesis. Nature 397, 315–323. 10.1038/16852 9950424

[B22] Lillis T. Veis A. Sakellaridis N. Tsirlis A. Dailiana Z. (2019). Effect of clopidogrel in bone healing-experimental study in rabbits. WJO 10, 434–445. 10.5312/wjo.v10.i12.434 31908992 PMC6937425

[B23] Lindemann S. Tolley N. D. Dixon D. A. McIntyre T. M. Prescott S. M. Zimmerman G. A. (2001). Activated platelets mediate inflammatory signaling by regulated interleukin 1beta synthesis. J Cell Biol. 154, 485–490. 10.1083/jcb.200105058 11489912 PMC2196422

[B24] Mao J.-T. Lai J.-N. Fu Y.-H. Yip H.-T. Lai Y.-C. Hsu C.-Y. (2022). Protective effects of higher exposure to aspirin and/or clopidogrel on the occurrence of hip fracture among diabetic patients: a retrospective cohort study. Biomedicines 10, 2626. 10.3390/biomedicines10102626 36289888 PMC9599449

[B25] Ono T. Hayashi M. Sasaki F. Nakashima T. (2020). RANKL biology: bone metabolism, the immune system, and beyond. Inflamm. Regen. 40, 2. 10.1186/s41232-019-0111-3 32047573 PMC7006158

[B26] Rezende E. Bradaschia-Correa V. Siviero F. Ambrosio L. M. B. Arana-Chavez V. E. (2017). Effects of bisphosphonates on osteogenesis and osteoclastogenesis signaling during the endochondral ossification of growing rats. Cell. tissue Res. 368, 287–300. 10.1007/s00441-017-2574-3 28220293

[B27] Sadeghi-Ghahrody M. Yousefi-Malekshah S. H. Karimi-Sari H. Yazdanpanah H. Rezaee-Zavareh M. S. Yavarahmadi M. (2016). Bleeding after tooth extraction in patients taking aspirin and clopidogrel (Plavix®) compared with healthy controls. Br. J. Oral Maxillofac. Surg. 54, 568–572. 10.1016/j.bjoms.2016.02.036 26975576

[B28] Vestergaard P. Steinberg T. H. Schwarz P. Jørgensen N. R. (2012). Use of the oral platelet inhibitors dipyridamole and acetylsalicylic acid is associated with increased risk of fracture. Int. J. Cardiol. 160, 36–40. 10.1016/j.ijcard.2011.03.026 21463909

[B29] Vieira A. E. Repeke C. E. Ferreira Junior S. B. Colavite P. M. Biguetti C. C. Oliveira R. C. (2015). Intramembranous bone healing process subsequent to tooth extraction in mice: micro-computed tomography, histomorphometric and molecular characterization. PLoS One. 10, e0128021. 10.1371/journal.pone.0128021 26023920 PMC4449187

[B30] Wancket L. M. (2015). Animal models for evaluation of bone implants and devices: comparative bone structure and common model uses. Vet. Pathol. 52, 842–850. 10.1177/0300985815593124 26163303

[B31] Wei J. Wang J. Gong Y. Zeng R. (2015). Effectiveness of combined salmon calcitonin and aspirin therapy for osteoporosis in ovariectomized rats. Mol. Med. Rep. 12, 1717–1726. 10.3892/mmr.2015.3637 25891179 PMC4464425

[B32] Xie H.-G. Zou J.-J. Hu Z.-Y. Zhang J.-J. Ye F. Chen S.-L. (2011). Individual variability in the disposition of and response to clopidogrel: pharmacogenomics and beyond. Pharmacol. Ther. 129, 267–289. 10.1016/j.pharmthera.2010.10.001 20965214

[B33] Xin J. Zhan X. Zheng F. Li H. Wang Y. Li C. (2023). The effect of low-frequency high-intensity ultrasound combined with aspirin on tooth movement in rats. BMC Oral Health 23, 642. 10.1186/s12903-023-03359-3 37670292 PMC10478369

[B34] Yang X. Li J. Hu D. Chen J. Li Y. Huang J. (2016). Predicting the 10-year risks of atherosclerotic cardiovascular disease in Chinese population: the China-PAR project (prediction for ASCVD risk in China). Circulation 134, 1430–1440. 10.1161/CIRCULATIONAHA.116.022367 27682885

