# Corrigendum: Differential effects of clopidogrel and/or aspirin on the healing of tooth extraction wound bone tissue

**DOI:** 10.3389/fphys.2024.1522884

**Published:** 2024-11-12

**Authors:** Jiaping Wang, Juan Lin, Xin Song, Mengting Wang, Yan Chen, Ning Luo, Xin Wu

**Affiliations:** ^1^ Nanjing First Hospital, Nanjing Medical University, Nanjing, China; ^2^ Department of Stomatology, Nanjing First Hospital, Nanjing Medical University, Nanjing, China

**Keywords:** clopidogrel, aspirin, tooth extraction wound, osteogenesis, osteoclast, drug combinations

In the published article, there was an error in **affiliation** [2]. Instead of “Nanjing First Hospital, Department of Stomatology, Nanjing, China”, it should be “Department of Stomatology, Nanjing First Hospital, Nanjing Medical University, Nanjing, China.”

The authors apologize for this error and state that this does not change the scientific conclusions of the article in any way. The original article has been updated.

